# Small Magnetic Sensors for Space Applications

**DOI:** 10.3390/s90402271

**Published:** 2009-03-30

**Authors:** Marina Díaz-Michelena

**Affiliations:** Payloads and Instrumentation Area, INTA-Instituto Nacional de Técnica Aeroespacial, / Ctra. de Torrejón a Ajalvir km 4.2, Madrid, Spain; E-Mail: diazma@inta.es; Tel. +34-91-520-1183; Fax: +34-91-520-1065

**Keywords:** Miniaturized magnetic sensors, space magnetometers, AMR-Anisotropic MagnetoResistance, magnetic COTS-Components Off-The-Shelf

## Abstract

Small magnetic sensors are widely used integrated in vehicles, mobile phones, medical devices, etc for navigation, speed, position and angular sensing. These magnetic sensors are potential candidates for space sector applications in which mass, volume and power savings are important issues. This work covers the magnetic technologies available in the marketplace and the steps towards their implementation in space applications, the actual trend of miniaturization the front-end technologies, and the convergence of the mature and miniaturized magnetic sensor to the space sector through the small satellite concept.

## 1. Introduction

Curiosity and desire for knowledge about the universe seems to be something inherent in human beings. Since ancient times, the thirst for learning has gone beyond the outer atmosphere and into space. It was however, not until October 4^th^ 1957, with the placement of the first artificial satellite in Earth orbit that man was able to study space *in situ*.

Moving into space implies a constant challenge for technology, not only in terms of the technical requirements for particular devices but also because of the need to adapt technology to the extreme and often hostile environment in space. The upscreening of technology for the extreme conditions in space, in combination with the relatively small market constituted by space applications, often results in a huge increase in the cost of each component. Roughly, it can be said that each degree of qualification achieved (considering commercial, military and rad-hardened components for instance) results in an increase in the final price by one order of magnitude.

In the early stages of space exploration, coinciding with the Cold War, a huge budget was devoted to upscreening and testing of electronic components for space applications. This budget however, suffered a drastic reduction during the nineties. Apart from this, changing political agendas and missions as well as the diversification of the field is making it hard to continuously devote a high budget to all technology options.

One of the consequences of the end of the Cold War and the divergence of funding away from the space sector was the concept of the modern small satellite. As it was no longer possible to maintain a number of costly traditional missions a new philosophy of mission was needed. The slogan ‘Faster, Better, Cheaper’ (FBC) was coined by NASA and Aerospace Corporation [1]. This new philosophy consisted in simple and often small satellites developed in a short time and with commercial but highly functional components. This was possible because of the great advances made in semiconductors and integration techniques (VLSI-Very Large Scale Integration) in the last part of the century. The reduction of mission cost was achieved partly by focusing on reduced weight to minimize launch costs and partly by reducing the price of the different components. In contrast, the risk was increased.

Besides being small, cheap and with a relatively low weight, thereby contributing significantly to the reduction of mission costs, the commercial components, named COTS – Commercial Off-The Shelf – also had the advantage that delivery time was shortened. This had an important impact on the calendar of the missions as the functionality of the components could now be checked even with the very early models (EM-Engineering Models or even BB-Bread Boards). As a result a whole line of COTS upscreening and validation groups emerged in the working teams of all space agencies. In the following sections this work is focused on magnetic COTS and other small magnetometers, the combination of these sensors with a reduced front-end, and their application for space purposes.

## 2. Potential magnetic sensors for space applications

**Figure 1.**
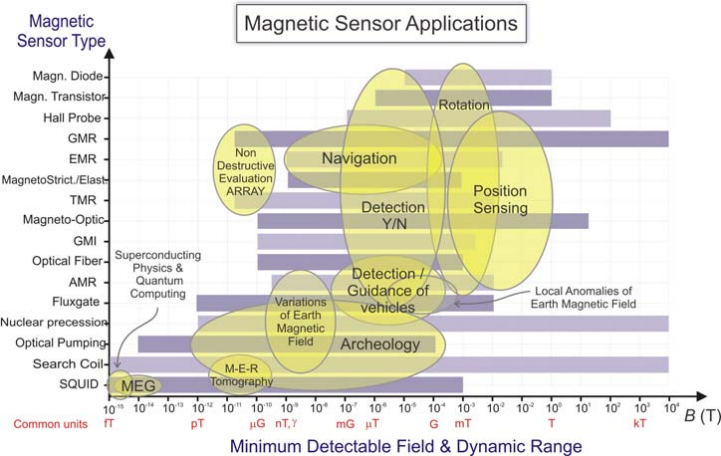
Magnetic Sensors Technologies: Magnetic properties (dynamic ranges and detectivities) and applications.

As happens in almost every technological niche on ground [2], magnetic sensors are useful for many applications in the space sector [3]. Though the most representative application is the in-orbit measurement of the magnetic field, there are some others as magnetic encoders [4], angular and position sensors [5] and magnetometers or gradiometers for planetary magnetometry. Since magnetic applications are so varied, the choice of magnetic sensor can be a difficult task. Figure 1 represents in a graph the panorama of the different magnetic sensors: the most representative technologies used for magnetic sensing are represented as a function of their magnetic characteristics: minimum detectable field and dynamical range. The applications have been depicted in Ben diagrams intersecting the bars of the technologies which can be used for the particular application.

This work is focused on potential COTS and small sensors for space measurements of the magnetic field or magnetic gradient. The magnetic field in-orbit can be measured for geomagnetic measurement purposes, or also inversely, to determine the relative orientation of a spacecraft in the geomagnetic field. This is the purpose of magnetic sensors in ACS – Attitude Control Systems [6]. In some missions measurement of the gradient of the field is also needed.

In general terms the requirements of the magnetometers used for geomagnetic field mapping are very strict. These magnetometers are required to measure the vector and scalar magnetic fields of the Earth with resolutions typical of the degrees 40 to 60 of the harmonics expansion of the field [7], the variations due to the ionospheric interaction and other perturbations [8–10]. The sensor technology par excellence for measuring the Earth magnetic field vector is the fluxgate because it is the best trade-off between resolution, stability and power consumption, mass and volume, and also some scalar magnetometers have been used to measure the intensity of the field or to complement the measurement obtained with fluxgates.

Fluxgates are based on the change of magnetic reluctance of a ferromagnetic core when it is driven by an ac saturating field in the presence of a magnetic field. The driving field is provided with the so called primary coil and the changes in the reluctance are measured by means of the secondary coil. These sensors are able to measure magnetic fields ranging from the mT to the tens of pT and in the range of frequencies from dc to the order of the operation frequency (tens of kHz). Fluxgates for Geomagnetic field mapping are usually sophisticated instruments manufactured *ad hoc*, precisely oriented (in the order of arcsec) with dynamical ranges of ± 64000 nT, bandwidths in the order of tenths of kHz (due to the relatively slow variations of the Earth magnetic field), resolutions in the order of tenths of nT to pT with long-term stability (1 nT in the whole range of temperatures of operation) [11] and low noise density (several pT/√Hz @ 1Hz). The main drawbacks of fluxgates are the high mass and power consumption. Typical mass of this kind of instrument can be in the range of 0.5 kg and 2 W of power consumption. Besides, both the reduction of mass and power decrease sensitivity and stability of the sensors. Thus, fluxgates should be used in those missions where volume and mass savings are not a priority, only moderate requirements.

Scalar magnetometers can be used alone (for isodynamical charts) or in combination with vector magnetometers for their calibration (absolute measurement). Proton precession (based on the principle that protons spin axis is aligned with the magnetic field), Overhauser (based on a quantum physics effect in hydrogen atoms), helium and cesium vapor magnetometers (based on the quantum mechanic change of absorption of the gas) are examples of scalar magnetometers. All of them are absolute sensors of the magnetic field and can improve one order of magnitude the resolution of the fluxgates.

The first mission with a magnetometer onboard was the Sputnik 3 (1958), which carried a fluxgate. There were also fluxgate magnetometers in the Lunik 1 and 2 (1959) devoted to the measurement of the magnetic field of the Moon. In the American side Ranger 1 and 2 (1961) had rubidium scalar magnetometers and the Mariner 4 (1964) to Mars and the Mariner 5 (1967) to Venus carried helium magnetometers.

Explorer 10 (1961) already used a combination of magnetometers separated a certain distance (dual technique): a rubidium scalar magnetometer and a fluxgate to measure the interplanetary field. With this technique the contribution of the platform to the magnetic field can be compensated. Besides scalar a vector magnetometers have complementary advantages. For example scalar ones can be used for calibration and vector ones can give more information about the sources of the field. This solution has been adopted by the big geomagnetic missions like MAGSAT (1979), which carried a cesium scalar magnetometer and a fluxgate vector sensor [12], Ørsted (1999) [13] and CHAMP (2000) with an Overhauser scalar and a fluxgate vector magnetometer, SAC-C (2000) with a helium magnetometer and a fluxgate [14], ST-5 [15] or the future SWARM in which the magnetic field will be not only measured at different points but also at different moments.

The Attitude Control System (ACS) of a spacecraft is that devoted to determine and control the orientation of the satellite. That is to sense and correct the relative orientation of the satellite within an inertial reference frame. Attitude determination for a spacecraft is in general a difficult task since it is continuously changing. To determine and control the attitude, spacecrafts normally use several sensors that work in a closed loop with actuators or torquers for the correction of the orientation of the spacecraft. These torquers can also be of many types: gas propulsion thrusters, magnetocoils, gravity gradient torquers, inertial wheels, gyros, etc.

In the ACS applications missions incorporating an optical camera, of Earth observation, military missions, or missions devoted to the development of accurate measurements of the geomagnetic field, like the ones mentioned above, have strict pointing needs respect to an inercial frame or reference and thus, (solid state) magnetic sensors, which can only achieve accuracies in the determination of 0.5°, are normally replaced by more accurate devices as star sensors (arc sec accuracies) together with accurate inertial sensors (gyroscopes) for a continuous quantification of the deviations in the attitude.

But there are other missions like some communications satellites, even with omnidirectional antennae, test-beds for technological experiments, etc, with lower requirements in which other alternatives can take place. In these missions the ACS requirements are more relaxed and they often use a combination of sensors for the determination of the attitude. This combination can include a magnetic sensor and other sensors as solar, planetary limb or gravity vector sensors as measuring elements. As actuators they can use for instance magnetic torques. For this kind of application other magnetic sensors apart from fluxgate are potentially good candidates.

Micro, nano, picosatellites and some minisatellites are in general representative examples of this situation. Requirements of the different magnetic applications are often relaxed, and have a philosophy headed to save mass and volume.

In these frames, commercially available technologies (COTS) [16] like AMR-Anisotropic MagnetoResistance, a mature technology, and the brand new GMR-Giant MagnetoResistance, MI-MagnetoImpedance and TMR-Tunnel MagentoResistance or SDT-Spin-Dependent Tunnel technologies (Table 1), can be plausible alternatives for magnetic purposes providing in most of the cases a miniaturized solution [17, 18]. This reason together with the lower price compared to rad-hardened components, and high functionality of the COTS sensors, motivate their use in space.

Among the sensors in Table 1, AMR sensors are the ones with a longer commercial history after the fluxgates. They achieved a high maturity level at the end of the nineties, as was demonstrated in relevant environments like, for instance, the automotive and the mobile communications sectors. The effect, discovered in 1857 by William Thomson (Lord Kelvin), consists in the change of electrical resistance (in the order of 3 %) in a magnetic material as a response of a variation in the environmental magnetic field [19–22]. To date AMRs have been used as magnetic field and current sensors, encoders, position and angular sensors, etc, in many on-ground applications. Commercial AMR sensors of the HMCxxxx series produced by Honeywell typically have dynamic ranges of hundreds of μT and resolutions in the order of 1 nT (detectivities in the order of nT/Hz^1/2^ for frequencies higher than 10 Hz [23]), with sensitivities in the order of 10 mV/(mT V_bridge_) with Set/Reset pulses [24]. They can measure fast variations in the magnetic field (bandwidth: 5 MHz) and they are silicon compatible, but they are highly temperature dependent. Thus, while they can operate over a huge temperature range (−55°C up to 155°C), their temperature sensitivity coefficients can be as high as 1 ‰/°C. They also display some variation in the offset with the temperature, although this is much less significant in general terms [23, 25]. The variation of the behaviour with temperature often is solved using Pt resistors glued to the magnetometer body.

This problem with the temperature is also a drawback in the relatively recently discovered (1988) GMR sensors. A GMR element consists of a trilayer or multilayer structure with two or more magnetic layers, each separated by a non-magnetic layer. Due to spin-dependent scattering of the conduction electrons, the resistance is maximum when the magnetic moments of the layers are antiparallel and minimum when they are parallel (the variation being as high as 20 %) [26–31]. In contrast to AMR (which has hysteresis in the order of 1 ‰ full scale), GMR devices present high hysteresis, up to 10 %, and thus their use for low magnetic field sensing is not trivial, and they were mainly used for reading heads in magnetic recording systems, i.e. hard disks [32]. However, GMR devices can also be used for sensing by means of a suitable resetting and biasing mechanism [33,34], achieving in the end good sensing properties and repeatability with the drawback of a higher power consumption (0.5 W). Since 1995 there have been commercial GMR devices devoted to other kinds of magnetic applications such as position and speed sensing and detection for medical and automotive solutions. GMR based sensors by NVE and Hitachi Metals have sensitivity values of 10–100 mV/(mT V_bridge_), ranges of up to 1 mT and minimum detectable fields in the order of tenths of nT [35].

More recently structures in which an insulating layer (instead of a conductive layer) separates two magnetic layers have been studied [36–41]. In these structures, electrical conduction between the two magnetic layers is allowed by quantum tunneling through the insulator. The tunneling current is a function of the relative magnetization direction in the two magnetic layers (SDT-Spin-Dependent Tunnel). The rotation of the magnetization of one of the layers caused by an external magnetic field can lead to relative resistance changes as high as 40 %. Sensitivities of SDT devices can easily be ten times those presented by AMR devices, depending on the composition and method used for the antiparallel disposition. The VLSI developed for these structures makes it possible to use them in array for high resolution (50 μm) mapping [42].

The next step in the development of magnetic sensors was the integration of sensing elements with MEMS-Micro-Electro-Mechanical Systems technology. This was the approach used by the Japanese companies Aichi Steel Corp and Aichi Micro Intelligent Corp who developed a MI-based sensor with a 1 nT resolution using MEMS technology [43]. The MI effect consists in the variation of the penetration depth of a ferromagnetic material when the magnetic field changes.

Finally there are groups that have developed real MEMS-based magnetic sensors [44] like resonant silicon magnetometers based on vibrating cantilevers [45–49] and torque magnetometers [50]. These sensors, which are not COTS, detect mechanical forces or torques on thin magnetic films deposited on a cantilever by capacitive, inductive or optical methods [51]. This kind of device is highly sensitive being able to measure magnetic moments in the order of 10^−17^ Am^2^ (and 10^−23^ Am^2^ at cryogenic temperatures). However these techniques require very controlled set-ups that cannot be easily achieved in the normally noisy and changing environments of spacecrafts.

Aside from the magnetic head of the sensors, their front-end has also experienced a huge evolution lately. Until this century, electronic devices could have either analog or digital interfaces but in general the integrated front-end was composed of discrete ICs-Integrated Circuits. With the invention of the FPGA- Field Programmable Gate Array in 1984 by Ross Freeman, this technology of programmable logic was implemented in some magnetic sensors in laboratory developments and also in a commercial level. Nowadays FPGA and ASIC-Applied Specific Integrated Circuit compete for miniaturization of the conditioning electronic of the sensors offering a low power solution. The ASIC option seems to be, for instance, the approach of Honeywell for the AMR sensors for mobile phone technology accurate electronic compassing, magnetometry, telematics, vehicle detection, position sensing and security systems. Both have advantages and disadvantages. FPGAs are more versatile but there is only a few work done with analogic FPGAs. Besides, so far the density of ASICs is higher to that of FPGAs. What is clear is that this line of miniaturization is the line of the future for the conditioning electronic of both COTS and highly qualified devices.

## 3. Adaptation of COTS for space applications

As described in the section above, both the functionality and integration of magnetic sensing technologies are continuously evolving. Moreover, as electronic devices and sensors are becoming essential parts of our daily lives and as users test them exhaustively the technology undergoes a continuous on-ground qualification process by which reliability is increased tremendously.

By the NASA nomenclature [52,53] of TRL – Technology Readiness Level - a systematic metric / measurement system that supports assessments of the maturity of a particular technology and the consistent comparisons of maturity between different types of technologies, and considering that a relevant environments is not restricted to the space environment, the TRL of a device that has been on the market for one year can be considered TRL 6 or 7: a demonstration in a relevant environment has been performed. Regarding magnetic sensing technologies, fluxgate, AMR and Hall effect vector sensors, and proton, helium or cesium scalar magnetometers can be considered mature sensors. The GMR, MI and SDT sensors described above, can be considered to be at a lower maturity stage. Besides, fluxgate sensors and several scalar sensors have been widely used for flight achieving in their missions a TRL 9. The key question is how to increase the maturity level of the alternative COTS sensors.

Keeping in mind the philosophy behind modern satellite design and the resources restrictions of some projects, very small sensors with a medium-to-high maturity level can be considered good candidates for space, the jump to space being achieved when it is demonstrated that the sensors can withstand the conditions in space for the life of the mission. The space environment, with extreme vacuum, temperature and radiation conditions, is highly hostile compared to that on the surface of the Earth. Besides, the satellites are exposed to strong mechanical stresses during the launch. Sensors to be used in flight need to be tested under these conditions. The upscreening for space qualification is a long and difficult process for magnetic sensors. Though the upscreening is usually conceived for each particular mission, in general terms the procedure consists of a sequence of thermal, vacuum, live cycle and radiation tests devoted to check the integrity of the sensors.

Most of the commercial components have a plastic package. Some plastics can outgas, thereby staining optical surfaces, depositing wherever and possibly causing serious damages. To avoid this, it is essential to define an outgasing test for the COTS with plastic package susceptible to outgas, before they fly. Fluxgates for flight usually have ceramic military packages, though there are some with plastic packages too. But the truth is that many components with plastic package do not outgas. For instance, some tests have been developed for AMR sensors of the HMC series by Honeywell showing that they do not present outgasing [54]. But the vacuum can also cause other damages like cracks in the components if for example bubbles were formed during the manufacture.

The temperature is another issue that needs to be taken into account. Temperature in the solar system ranges from temperatures as high as 485 °C on Venus, due to the greenhouse effect produced by its dense atmosphere of carbon dioxide, to −200 °C for the outer planets, with intermediate temperatures as high as 400 °C in Mercury, and as low as −130 °C in Mars and Jupiter or −190 °C in Saturn. For satellites orbiting around the Earth the temperatures are not that extreme, but they can be far outside the commercial temperature range (0°C, 70 °C) and even the industrial range (−40°C, 85 °C). In some of the small platforms passive thermal control can achieve and maintain a moderate temperature environment of around 50 °C. In other cases, a temperature cycling at the operating and storage temperatures is developed for the COTS. In this cycling test the speed of the variation of the temperature needs to be representative but fast enough so as to simulate the total life of the mission. Other temperature tests are the thermal shock resistance test and a working test when sensors need a driving excitation. For instance in the case of fluxgates it is convenient to cycle during a long time the driving current and in the AMR sensors the Set/Reset current pulses to guarantee the repeatability and maximum sensitivity and because in the range of temperature the variation of the electrical resistance of the coils can be considerable.

The temperature can also affect the mechanics of the device leading to cracks and movements of the lithographied parts. Often materials with compensated thermal expansion coefficients have to be used for the assembly to avoid stresses or changes of shape of the elements that eventually can influence the behaviour of the sensors or break them. But attention needs to be paid too when using metallic surfaces because thermal gradients can generate thermoelectric currents that disturb the measurement.

Another problem with in-orbit missions is the radiation. The sources of radiation are diverse. Apart from other missions to the giant planets, perhaps the most harmful for in-orbit missions and mostly in LEO-Low Earth Orbit, are the Van-Allen belts, where electrons and protons are trapped around the lines of the Earth magnetic field. These belts approach the surface of the Earth in the South Atlantic anomaly. But there is also radiation, which comes from the outer space, cosmic radiation, and solar flares associated to the maximums of activity in the solar cycles. The effects of radiation on the components can be divided into three main groups: TID - Total Ionizing Dose, DD - Displacement Damage and SEE - Single Event Effects.

The TID effect consists of the damage inflicted by the charging process caused by the accumulated dose in semiconductors and other components. Normally to simulate or study the influence of this kind of effect on the components, γ-ray irradiation campaigns are developed covering the expected total dose during the life of the missions. A reference for a three year mission in a polar LEO (700 km) can be 10 krad (with the typical aluminum shield of 1 mm-spherical).

DD is characterized by changes in the crystalline structure of a device produced when a high energy particle passes through it. This effect, mostly produced by protons, can be equivalent to TID and some times more significant than TID, like in some semiconductors.

The SEE consists of the damage caused generally by high energy particles when they pass through a material. These effects can be temporary or permanent. The way of testing components for SEE effects is through irradiation with high energy atomic nuclei and particles.

Regarding magnetic technologies, Hall sensors seem to be more sensitive to radiation damage than AMR sensors, for instance [55]. INTA, the Spanish National Institute of Aerospace Technology has tested both AMR and GMR COTS sensors with γ-ray with total doses higher than 10 krad and with 52 MeV protons with a flux of 2.5 × 10^8^ protons/cm^2^s achieving a fluence of 2 × 10^12^ p/cm^2^. The COTS sensors (AMR sensors of the family HMCxxxx by Honeywell and AAL002-02 GMR sensors by NVE) did not experience substantial damage [54].

In the case of magnetic devices, both electrical and magnetic properties have to be checked in the end of each test. This last part of the testing has to be done very carefully (using shielding chambers) as most of the thermal and vacuum chambers have ferromagnetic composition, which seriously influence the magnetic measurements [3]. In the case of these devices the reliability is conditioned to the magnetic cleanness of the pre and post test calibrations.

Although the sensor head technology is demonstrated to be robust, the associated front-end can be susceptible to damage by the factors mentioned above. To produce robust FPGAs and ASICs is one of the big challenges for space applications manufacturers.

## 4. Magnetic sensors for space missions. The implementation of magnetic COTS in spacecrafts

As it has been introduced, the history of the magnetic sensors for space starts with the Soviet Sputnik-3. Sputnik-3 was a 1.3 ton satellite devoted to researching the upper atmosphere and near space. The onboard instrumentation contained the first vector magnetometer: a three axis fluxgate magnetometer, which however did not succeed in the determination of the direction of the Earth magnetic field due to the uncertainty in the attitude of the spacecraft.

Triaxial fluxgate magnetometers have been widely used for monitoring the magnetic fields of the Earth and Moon (Luna 1, Luna 2, Pioneer Venus, Mariner 2, Venera 1, Explorer 12, Explorer 14, and Explorer 15). Explorer 33 already foresaw a boom for the magnetometer to avoid in part the contribution of the spacecraft to the total magnetic field in the orbit of the Moon. But this purpose was not achieved until Explorer 35 entered a stable orbit around the Moon.

Fluxgates have evolved with magnetic space missions [3]. The fluxgate technology has been improving simultaneously with the techniques of magnetic field sensing and now fluxgates are still the sensors that present the best magnetic properties for the measurement of the geomagnetic field. It has to be taken into account that an increase in the knowledge about the magnetic field of the Earth only can be achieved by means of very fine measurements since actual models of the field already reproduce the contribution of the core, the crust and the ionospheric phenomena to the global field, thanks to the data provided by POGO-Polar Orbiting Geophysical Observation missions (1967–1971), Magsat (1979), Astrid-2 (1998) [56], SAC-A (1998), Ørsted (1999), Komsat (1999), CHAMP (2000), ST-5 (2006) [15], etc.

It cannot be said that geomagnetic missions have generally followed the trend of the modern concept of small satellites, as almost all of these missions were medium-to-large satellites with Astrid-2 (with a mass of 30 kg) being the smallest amongst them. Besides, apart from the weight of the sensor, often it has to be taken into account the weight of the deployment system. MAGION-1, the first Czech MAGnetopheric and IONospheric satellite launched in 1978, and devoted to doing research on the magnetosphere and the ionosphere could have been considered an exception due to its smaller weight of 15 kg. This prism-shaped satellite of 300 × 300 × 150 mm^3^ also carried a vector fluxgate magnetometer for Earth magnetic field measurements. Regrettably, this mission was not very successful since a failure in the power system resulted in limited data for a certain period. It re-entered the atmosphere on September 11^th^, 1981 [57].

Nevertheless, the magnetometers for other on-board applications have been deeply affected by this trend towards miniaturization. In many missions the big box or boxes for the fluxgates (in most of the cases the sensor head is located in a different box than the front-end electronics) with a power consumption in the order of the Watt, are being replaced by smaller sensors that comply with the magnetic requirements. Actually, in some applications small sensors may be the better choice since they can be easily disposed in array [42] with temperature sensors for temperature compensation, thereby reaching a better trade-off between magnetic performance and spatial resolution [58].

This is illustrated for instance by the case of the LISA (Laser Interferometer Space Antenna) mission of ESA-European Space Agency. LISA is aimed at detecting gravitational waves generated by black holes. The measurement is to be performed by two test-masses floating inside a cavity in one of the Lagrange points, where the gravitational attraction by the Earth and the Sun are compensated and there is a nearly perfect gravitational free-fall. To do so LISA needs to be capable to distinguish among gravitational forces and other type of forces. Thus, the magnetic cleanness requirements are very strict with magnetic field lower than 10 μT and Power Spectral Density - PSD lower than 650 nT /√Hz, magnetic field gradient (proportional to the magnetic force) lower than 5 μT/m and PSD lower than 250 (nT/m)/√Hz. In the beginning of the project four fluxgates out of the cavity were to be used to measure the magnetic environment inside the cavity. Since the knowledge of the magnetic behaviour can be hardly deduced with this configuration, there is an actual trend to substitute the bulky fluxgates by an array of small solid state sensors temperature compensated [59].

But still the magnetic application where miniaturized sensors has been used is the ACS. As it has been said many satellites use magnetometers for attitude determination. Close to 63 % of the small satellites since the nineties used magnetic-based ACS or at least magnetic devices combined with sensors and actuators of another nature (GPS, Sun sensors, Earth sensors, gyrosensors, microvacuum arc thrusters, gravity gradient sensors, etc).

In the beginning, these satellites inherited the fluxgate magnetometers from the conventional missions but as soon as mass, volume and power savings became more important requirements, the use of COTS magnetic sensors became a normal practice.

The first microsatellite, according to the modern concept of small satellite, was the UoSAT-1 or OSCAR-9. OSCAR – Orbiting Satellite Carrying Amateur Radio – was the first program of modern microsatellites produced by the AMSAT-AMateur radio SATellite community. Their first launch (1961) consisted of a simple radio-satellite, but the subsequent missions have become more and more sophisticated with time.

UoSAT-1 was the first experimental satellite from the University of Surrey in the UK. The 52 kg and 420 × 420 × 740 mm^3^ platform, launched in 1981 by a Thor Delta launcher from the Vandenberg Air Force Base, carried research, technology demonstration and educational payloads.

The magnetometer of UoSAT-1 consisted in a three-axis fluxgate that provided vector measurements of the Earth magnetic field in a boom at a rate of 6.25 vectors per second. Each orthogonal component had a range of around 33 μT and a resolution of 2 nT. Shortly afterwards and motivated by the success of UoSAT-1, the group developed in just half a year the UoSAT-2, a 45.5 kg and 350 × 350 × 650 mm^3^ platform to be launched in 1984. It was thanks to these two satellites that the University of Surrey became a world leader in the small satellites field by means of the SSTL-Surrey Satellite Technology Ltd spin-off company.

The example was followed by other universities leading to smaller and smaller satellites like the OSCAR-18 (WO18, WeberSat), a 16.03 kg and 150 × 150 × 150 mm^3^ size platform, developed by Weber State University in Utah in 1990 [60–62].

The ADCS-Attitude Determination and Control System of OSCAR-18 comprises a horizon sensor using two photodiodes with 22 ° field of view and a fluxgate magnetometer as sensing elements and permanent magnets, hysteresis rods, and black/white painted antennas for spin control (similar to a photometer) as actuators. Besides, it also carried, as payloads, a piezoelectric particle impact detector, which measures the micrometeorite environment and a light spectrometer using a CCD - Charged-Coupled Device by NEC, which measures the spectrum of sunlight reflected from the atmosphere. The satellite suffered an apparent radiation induced computer failure on August 23^rd^, 1997 and it did not recover from the failure until November 1997.

**Figure 2.**
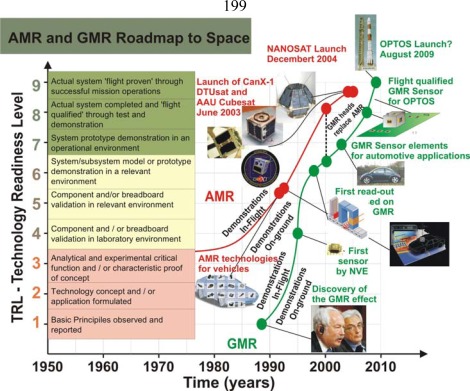
The graph represents the roadmap for space of AMR and GMR technologies. In the horizontal axis it is represented the time and in the vertical axis the Test Readiness Level. Below the curves some of the on-ground demonstrations are shown; above the curves the in-flight demonstrations are shown.

The jump to COTS technologies for the ACS of these small satellites had to wait until the new millennium. At that moment there were fluxgates off-the-shelf but also there were solid state sensors off-the-shelf, which had already been exhaustively tested on-ground applications (TRL higher). Figure 2 shows the roadmap of AMR and GMR magnetic sensors. AMR technology was already mature in the nineties and its use on Earth was widely spread. GMR effect in contrast, was discovered in 1988 and has experienced a fast industrial development together with the “VLIS” techniques. In 2003 several missions with AMR COTS were launched: the American ION-F [63], the Canadian CanX-1 [64,65], the Danish DTUsat [66] and AAU Cubesat [67], etc. Some of these spacecraft were based on an Off-The Shelf structure, the Cubesat concept [68], developed by Stanford University and Santa Clara University in 2000 and inspired by the FBC concept.

ION-F – the Ionospheric Observation Nanosatellite Formation of the three satellites [69]: DawgStar (University of Washington) [70], USUSat (Utah State University) [71] and HokieSat (Virginia Technological University) [72], all of them between 10 and 15 kg, used the HMC2003 three-axis AMR magnetic COTS sensor by Honeywell for the measurement of the Earth magnetic field up to a 2 ° accuracy together with Fuga 15d CCD cameras by Vector International as Earth′s horizon sensors (9 ° accuracy), solar arrays by TECSTAR as Sun sensors (10 ° accuracy) and a three solid-state quartz rate sensor by BEI Systron Donner for the measurement of the angular velocity (0.004 °/s accuracy). The same sensor was used on board CanX-1, devoted to space-testing of key technologies for future missions.

In the same line, but with a different approach were the Danish student projects of cubesat-based platforms - DTUsat by the Danish Technical University and AAU Cubesat by Ålborg University. DTUsat used four (for redundancy) one-axis HMC1021 magnetic sensors and AAU Cubesat used the one-axis HMC1001 in combination with the two-axis sensor HMC1002 for a vector measurement of higher resolution.

Many other missions have successfully used these AMR analog sensors in the following years like the Spanish NANOSAT (2004) [73], the Norwegian NCubes (2005), the Japanese CUTE 1.7 (2005) or the American ION (2005).

**Figure 3.**
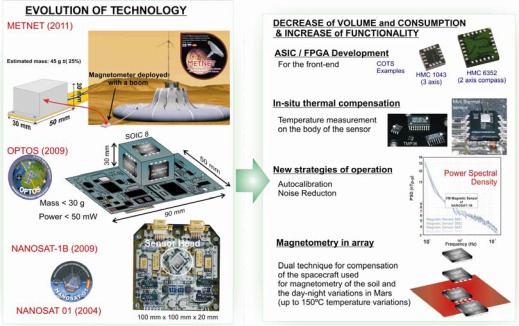
The graph represents the evolution of AMR-based sensors of INTA. In the right hand side of the picture the techniques used in the evolution are listed. In the left hand side of the picture the time evolution of the sensors is shown, starting in 2004 with NANOSAT-01 and the actual goal for the MetNet Precursor magnetometer.

GMR sensors have not been flown yet but INTA, the Spanish National Institute of Aerospace Technology, is working in the adaptation of a miniaturized GMR three axis sensors for a triple-cubesat called OPTOS (to be launched in 2009) [34]. In this line, INTA is also working in the validation of a MI-based three-axis experimental sensor for a nanosatellite called NANOSAT-1B (to be launched in the end of this year) respectively. Both satellites, OPTOS and NANOSAT-1B will have quasi-polar low Earth orbits (650 km height approximately) with a maximum intensity of the magnetic field of 48.75 μT (at −60° latitude). NANOSAT program is the technological experimentation platform of INTA. OPTOS is a picosat of 3 kg for the demonstration of a distributed wireless on-board computer. The resolution required for the experimental magnetic sensors on-board NANOSAT-1B and OPTOS is 10 nT. Figure 3 shows the evolution of AMR-based magnetometers developed by INTA. The institute started flying AMR sensors as ACS in the NANOSAT program because they offered a good trade-off between power, mass, volume and performance and the developments have been gradually miniaturized. The actual goal is to develop double AMR vector sensor with an ASIC as front-end for MetNet Precursor mission. With a total envelope of 135 g for three payloads, this magnetometer will allow us to do some preliminary *in situ* magnetometry even though some of the scientific objectives could be not reached.

In the implementation of VLSI techniques some previous work has already been done with fluxgates. For example SMILE-Small Magnetometer In Low-mass Experiment consists in the development of a miniaturized fluxgate with FPGAs developed in the School of Electrical Engineering of the Royal Institute of Technology [74] and the Magnetometer Front-end ASIC (MFA), developed by the Space Research Institute (Austrian Academy of Sciences) in Austria [75]. Regarding the implementation of these VLSI techniques for COTS it can be mentioned the launch of the German cubesatellite Compass-1 on April 28^th^ with the PSLV - Polar Satellite Launch Vehicle (PSLV) from the Satish Dhawan Space Centre in India. The satellite, devoted to the technology demonstration of a miniature GPS receiver, and a transceiver for fast RF communication, uses an ASIC integrated two-axis COTS magnetometer HMC6352 [76] by Honeywell, together with Sun sensors and magnetotorquers for the ACS. Nowadays the implementation of FPGAs and ASICs in the front-end of magnetometers implies an increase of the cost and the complexity of the development of the sensors in general and in particular for space applications. But due to the fact these circuits are being more demanded by the market, it can be said facing the future that the use of FPGAs and ASICs in the spacecrafts is likely to be the trend for the actual and future concept of satellites: the sensor-satellites and even the satellite-on-a chip.

To summarize, so far the two trends of magnetic sensors coexist in the space panorama for different requirements: the combination of two magnetometers manufactured *ad hoc* for geomagnetic field mapping and magnetic COTS sensors. Miniaturized magnetic sensors (miniaturized fluxgates, AMR sensors, etc) seem to have poorer stability and less resolution than the bulky fluxgates manufactured *ad hoc* for in orbit geomagnetic purposes but in contrast, COTS miniaturized sensors offer a lighter, low power and reasonable good performance solution for many cases.

The present evolution towards miniaturization can be considered to aim for two main objectives: on the one hand, a huge effort is being done in the implementation of FPGAs and ASICs in the front-end of the magnetic sensors and on the other hand, some qualification process are on the road for the adaptation of brand new technologies to space.

## 5. Conclusions

In the evolution from the conventional satellites to the satellite-on-a-chip concept it seems to be that two lines of magnetic sensors are developing in parallel depending on the particular application. In the use of magnetic sensors for geomagnetic field mapping, the combination of fluxgates vector sensors and scalar sensors deployed in a boom are the best trade-off between performance and resources consumption. For other applications like magnetic-based ACS-Attitude Control System when the accuracies of pointing requirements are medium-to low, mature off-the-shelf technologies such as AMR-Anisotropic Magnetoresistance and miniaturized commercial fluxgate sensors offer a good solution. For gradient measurements when accurate mapping of a region has to be done, miniaturized sensors like AMR or SDT-Spin-Dependent Tunnel sensors should be used, since they can be configured in arrays offering better spatial mapping. Nevertheless, these miniaturized sensors should be used when high spatial resolution is needed. The use of brand new technologies in the space requires a tested reliability of the technology in the market and the development of a qualification test adapted to the conditions and the life-time of the mission. When technologies are not mature, the cost of the adaptation for space is very high and normally unaffordable by the mission but effort devoted to watch the emerging transducer for a new miniaturized but competitive magnetic sensor technology is very much worthy.

Regarding the front-end electronics of the sensors it seems to be a convergence of both lines of magnetic sensors to the use of VLSI- Very Large Scale Integration technologies: nowadays FPGAs-Field Programmable Gate Array and ASICs-Applied Specific Integrated Circuit technologies.

## Figures and Tables

**Table 1.** Magnetic properties and approximate price of some commercial magnetic sensors suitable for space applications.

**Technology**	**Comercial device**	**Magnetic Field Range (μT)**	**Resolution (nT)**		**Noise Density (nT/√Hz)**	**Sensitivity (mV/mT)**	**BW**	**Mass (g)**	**Price (€)**	**Observations**

**3-axis FG**	Small ones of several manufacturers	±64	< 0.1	5 V	<0.02 @ 1Hz	100000	> 5 kHz	< 100	< 250	Drift < 10 nT / year Front-End included
				1 Hz						

**AMR**	HMC1021	±600	8.5 @	5 V	2 @ 1 Hz	50 @ 5 V	DC-5MHz	< 0.5	24	Manufactured with Barber Pole biasing
				1Hz						

**GMR**	AAL002	1,5	10 @	5 V	1 @ 1 Hz	175 @ 5 V	>1 MHz	< 0.5	7	Needs biasing
				1Hz						

**SDT**	NVE	±200	1		1 @ 1 Hz	---	125 Hz	< 0.3	70	COTS not very widely spread yet

**MI**	AMI302	±1,000	10 @	5 V	5 @ 1 Hz	1,335 @ 3 V	1 kHz	< 0.5	70	Front-End included
				1Hz						
